# Attenuation of early liver fibrosis by herbal compound “Diwu Yanggan” through modulating the balance between epithelial-to-mesenchymal transition and mesenchymal-to-epithelial transition

**DOI:** 10.1186/1472-6882-14-418

**Published:** 2014-10-27

**Authors:** Xin Shen, Sisi Cheng, Yu Peng, Hongli Song, Hanmin Li

**Affiliations:** Department of Laboratory Medicine, Hubei University of Chinese Medicine, Wuhan, 430065 People’s Republic of China; Hepatic Disease Institute, Hubei Provincial Hospital of Traditional Chinese Medicine, Wuhan, 430061 People’s Republic of China; Department of Information Engineering, Hubei University of Chinese Medicine, Wuhan, 430065 People’s Republic of China

**Keywords:** Liver fibrosis, Diwu Yanggan, Epithelial-to-mesenchymal transition, Mesenchymal-to-epithelial transition, TGF-β1/BMP-7, Hedgehog signaling pathway

## Abstract

**Background:**

Diwu Yanggan (DWYG) is a Chinese compound herbal preparation which consists of five Chinese herbs. This study investigates the preventative effects of DWYG on liver fibrosis induced by carbon tetrachloride (CCl_4_) and explores its possible mechanisms of action.

**Methods:**

Liver fibrosis was induced in male Wistar rats by injecting a 50% CCl_4_/soybean oil solution subcutaneously twice a week for six weeks. After six weeks of treatment, serum aspartate transaminase (AST) and alanine transaminase (ALT) assay, liver tissue histological assessment and hepatic hydroxyproline assay were respectively carried out to examine the effects of DWYG on liver function and fibrosis degree. The impacts of DWYG on the expression levels of epithelial marker E-cadherin, mesenchymal marker Vimentin, transforming growth factor β1 (TGF-β1) and bone morphogenetic protein-7 (BMP-7) were further examined by quantitative real-time RT-PCR and Western blot analysis. In addition, the differences of Hedgehog (Hh) signaling pathway activity between DWYG-treated and DWYG-untreated fibrotic liver tissues were also evaluated by quantitative real-time RT-PCR and Western blot analysis.

**Results:**

Upon DWYG treatment, the serum levels of ALT and AST, hepatic hydroxyproline content and the degree of fibrosis in CCl_4_-induced fibrotic model rats were dramatically declined. In accompany with the alleviation of the degree of fibrosis, DWYG treatment provoked the reversal of epithelial-to-mesenchymal transition (EMT) to mesenchymal-to-epithelial transition (MET) in the fibrotic liver tissues, which was characterized with the up-regulation expression of E-cadherin and down-regulation expression of Vimentin. Furthermore, we observed that the expression level of TGF-β1 was reduced whereas the expression level of BMP-7 was enhanced in liver tissues of DWYG-treated rats, therefore the expression ratio of TGF-β1/BMP-7 was dramatically decreased compared to CCl_4_-induced fibrosis model rats. In addition, quantitative real-time RT-PCR and Western blot analysis demonstrated that after DWYG treatment the expressions of Hh ligand Shh, receptor Smo and Ptc, and transcription factor Gli1 in CCl_4_-induced fibrotic liver tissues were dramatically repressed.

**Conclusions:**

DWYG demonstrates therapeutic potential to prevent liver fibrosis by modulating the balance between EMT and MET through reducing the expression ratio of TGF-β1/BMP-7 and inhibiting the excessive activation of Hh signaling pathway.

## Background

Liver fibrosis is a wound-healing process that responds to diverse types of chronic liver injuries, such as viral infection, alcoholic, drug or chemical toxicity. The persistent activation of wound healing responses causes the unbalanced extracellular matrix (ECM) deposition and resolution, and could result in progression to cirrhosis, liver failure, and portal hypertension, which are often associated with considerable morbidity and mortality [1]. Emerging anti-fibrotic therapies are aimed at inhibiting the accumulation of fibrogenic cells and/or preventing the deposition of extracellular matrix proteins. Although many therapeutic interventions are effective in experimental models of liver fibrosis, their efficacy and safety in human are not always satisfactory [2]. This highlights the urgent need to both increase our understanding of the mechanisms of liver fibrogenesis and develop novel therapies to arrest or reverse the fibrotic process as even advance fibrosis is reversible [3, 4].

Recently, the role of epithelial-to-mesenchymal transition (EMT) in hepatic fibrogenesis as an evolving pathogenetic concept has drawn extensive attention. EMT is defined as the biological process by which cells gradually lose typical epithelial characteristics and acquire mesenchymal traits, which is of crucial importance not only during embryonic development but also in adult tissue remodeling [5]. In chronic liver diseases emerging evidences suggest that activated hepatic stellate cells were not the only key players in the hepatic fibrogenic process and that other cell types, either hepatic (i.e. hepatocyte, biliary epithelial cell and portal fibroblast) or extra-hepatic (bone marrow-derived cells and circulating fibrocytes) could contribute to this process through EMT [6–8]. Moreover, mesenchymal-to-epithelial transition (MET), as the reverse process of EMT, indicates the transformation of mesenchymal cells to acquire epithelial traits. MET has long been known to be of paramount importance in normal embryonic development, but has only recently been shown to represent an attractive concept in counteracting fibrotic damage in acute and chronic renal disease processes [9]. *In vitro* and *in vivo* studies have reported that the EMT-derived mesenchymal cells could be reverted to epithelial cells that ultimately become hepatocytes or cholangiocytes through the exogenous regulation of MET in liver fibrogenic injury [10, 11]. The important roles of EMT and MET in liver tissue injury and repair, in addition to their potential reversibility, made this trans-differentiation process a relevant and suitable target for anti-fibrogenic strategies in liver fibrosis. Furthermore, this trans-differentiation process is known to be tightly controlled by modulating factors, such as TGF-β1, BMP-7 and Hh signaling. Therapeutic manipulations of these known modulating factors have generally been demonstrated to influence liver regeneration and fibrosis in rodents [12].

Diwu Yanggan capsule (DWYG) has been invented as a new anti-fibrotic medicine, and consists of five Chinese medicinal herbal extracts. In clinical study, we observed that DWYG could have significantly hepato-protective effects on the patients with chronic hepatitis B infections as evidenced by the results of an obvious decrease in serum ALT and AST levels [13]. More importantly, the results of percutaneous liver biopsy demonstrated that DWYG could effectively decrease the degree of fibrosis in the patients with chronic hepatitis B infection, suggesting it might be an effective antagonist of liver fibrosis (data not shown). Following pharmacological studies showed that DWYG could decrease the serum levels of many cytokines in the patients with chronic hepatitis B infection, particularly TGF-β1 and IL-6 (data not shown). In addition, the active constituent of DWYG, such as curcumin, has already been reported to block the activation of Hh signaling pathway through inhibiting Shh expression [14]. However, the underlying therapeutic mechanisms of DWYG on liver fibrosis still remain obscure even though it has shown to provide clear therapeutic benefit.

Based on the above mentioned information, in this study we investigated the effects of DWYG on carbon tetrachloride (CCl_4_)-induced hepatic fibrogenesis during the initial phases in rats. Moreover, we clarified the impacts of DWYG on the trans-differentiation process of EMT/MET, and to further explore its potential modulation mechanism we analyzed the effects of DWYG on the activities of TGF-β1, BMP-7 and Hh signaling pathway, which contributed to explain its underlying therapeutic mechanism.

## Methods

### Characterization and preparation of herbal compound

**Figure 1:**
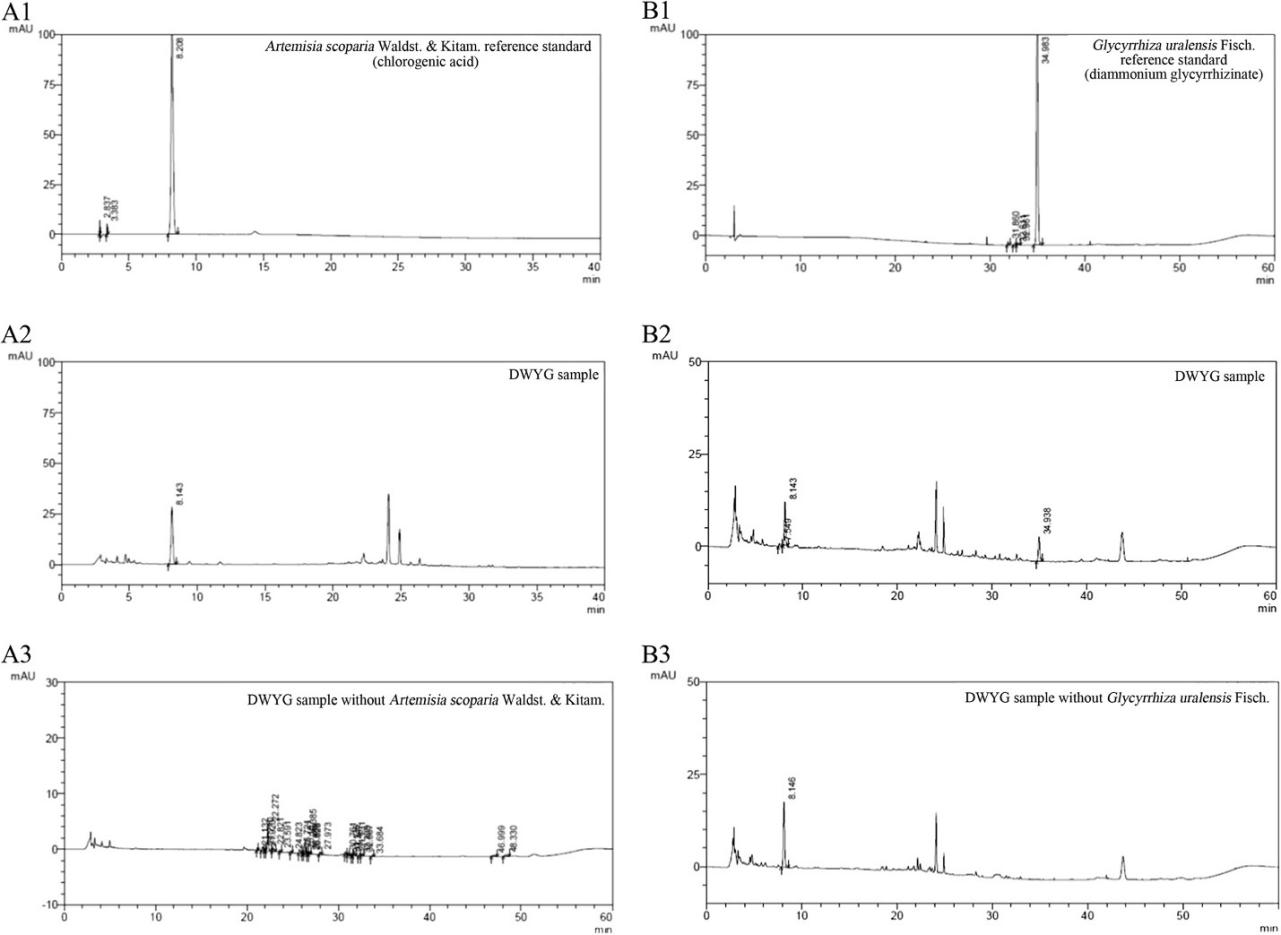
**Two representative components (Chlorogenic: A1-A3; Diammonium glycyrrhizinate: B1-B3) of DWYG capsules for quality control were determined by high-performance liquid chromatography (HPLC).**

The Chinese herbal medicine formula DWYG is a new drug authorized by the Hubei Food and Drug Administration (Grant No. Z20113160). The mixture includes five Chinese medicinal herbal extracts, whose proportions (w/w) are as follows: *Rehmannia glutinosa* (Gaertn.) DC. 20.0%; *Artemisia scoparia* Waldst. & Kitam. 33.3%; *Curcuma longa* L. 13.4%; *Schisandra chinensis* (Turcz.) Baill. 20.0%; *Glycyrrhiza uralensis* Fisch. 13.4%. The DWYG capsules used in this study with the same batch number (20120221) were provided by Traditional Chinese Medicine Preparation Room of Hubei Provincial Hospital of Traditional Chinese Medicine. Briefly, the DWYG capsules were prepared as follows: The decoction of *Rehmannia glutinosa* (Gaertn.) DC. and *Glycyrrhiza uralensis* Fisch., the coarse powder of *Schisandra chinensis* (Turcz.) Baill., *Artemisia scoparia* Waldst. & Kitam. and *Curcuma longa* L. were mixed together, then added 75% ethanol and extracted by reflux extraction three times. The ethanol extracts were filtrated, concentrated and removed ethanol by reduced pressure. The DWYG capsules were finally obtained by decompression drying and granulating of the above refined concentrates. In this study, DWYG capsule was suspended in normal saline and its final concentration was 36 mg/mL. Two representative components that might chemically represent characteristic components of herbal medicines for quality control were determined by high-performance liquid chromatography (HPLC) and shown in Figure 1.

### Animal experiments

Male Wistar rats (SPF class, weighing 200–250 g) were purchased from the Hubei Experimental Animal Center of the Chinese Academy of Sciences (Wuhan, China). All rats were housed with a 12 h-light–dark cycle and with water and standard chow *ad libitum*. The rats were randomly divided into three treatment groups: CCl_4_-induced model group (n = 6), DWYG-treated group (n = 6) and blank control group (n = 6). In the CCl_4_-induced model group, rats were treated with subcutaneous injections of CCl_4_ (suspended at 50% in soybean oil, 1 mL/kg) for 6 weeks; In the DWYG-treated group, besides the subcutaneous injections of 50% CCl_4_/soybean oil twice a week, rats were simultaneously treated with intragastric administrations of the solution of DWYG capsules (suspended in normal saline, 360 mg/kg) once a day for 6 weeks; and in blank control group, rats were treated with both subcutaneous injections of soybean oil (1 mL/kg, twice a week) and intragastric administrations of normal saline (the same volume as that of the solution of DWYG capsules given in the DWYG-treated group) once a day for 6 weeks. All rats were sacrificed and serum was collected for serum enzyme analysis, and then liver pieces were fixed in 10% neutral buffered formalin or snap frozen in liquid nitrogen for further analysis. The protocols of all animal experiments were approved by the Hubei Provincial Laboratory Animal Care and Use Commission.

### Determination of serum ALT and AST activities

Serum concentrations of aspartate transaminase (AST) and alanine transaminase (ALT) in different treatment groups were measured using rat ELISA kits commercially available from Shanghai Westang Bio-tech CO., LTD according to the manufacturer’s instructions.

### Hydroxyproline assay

Hepatic hydroxyproline content was quantified colorimetrically in flash frozen liver tissue samples of different treatment groups as described by Lee *et al*. [15]. Briefly, 30 mg liver tissue samples were hydrolyzed in 6 mol/L HCl at 120°C for 16 h. After centrifugation, the supernatant was removed and neutralized with 6 mol/L NaOH. The solution was oxidized with Chloramine T (Sigma-Aldrich Corp., St Louis, MO, United States) in acetate/citrate buffer, followed by the addition of Ehrlich’s solution. The final mixture was incubated at 60°C for 30 min and then at room temperature for 10 min. Absorbance was determined at 560 nm. The value of the hepatic hydroxyproline concentration was expressed as μg/g wet liver tissue.

### Liver histology and morphometry

Liver tissue samples of different treatment groups were fixed in 10% neutral buffered formalin, paraffin-embedded, and sectioned at 5 μm. For standard histology, liver sections were stained with Hematoxylin-eosin (H & E) and Masson’s trichrome and then analyzed blindly as described previously [16]. Collagen staining of liver sections with Masson’s trichrome was assessed by morphometric analysis (Image-Pro Plus 6.0 software, Media Cybernetics, USA). Ten randomly chosen × 20 fields/section were evaluated for each mouse.

### RNA isolation and quantitative real-time RT-PCR

Total RNA was isolated from the liver tissue samples of different treatment groups with TRIzol Reagent (Invitrogen) according to the manufacturer’s protocol. RNase-free DNase (Promega) was used to eliminate the contamination of genomic DNA. The first cDNA strand was synthesized by using a ReverTra Ace qPCR RT Kit (Toyobo, Japan), and then amplified using THUNDERBIRD SYBR qPCR Mix (Toyobo, Japan) and specific primers on the Mx3000P QPCR System (Stratagene, USA). Primer sequences are listed in Table 1 and the housekeeper gene GAPDH was used as an internal control. Target gene levels in the samples are presented as a ratio of levels in treated tissues to levels detected in corresponding control tissues according to the 2^-ΔΔC*t*^ method [17].

**Table 1:** **Primer sequences**

Gene	Forward sequence	Reverse sequence	Product size (bp)
E-cardherin	5′-GGGTTGTCTCAGCCAATGTT-3′	5′-CACCAACACACCCAGCATAG-3′	184
Vimentin	5′-AGATCGATGTGGACGTTTCC-3′	5′-CACCTGTCTCCGGTATTCGT-3′	205
TGF-β1	5′-GCTGAACCAAGGAGACGGAAT-3′	5′-CGGTTCATGTCATGGATGGTG-3′	143
BMP-7	5′-GAGGGCTGGTTGGTATTTGA-3′	5′-AACTTGGGGTTGATGCTCTG-3′	121
Shh	5′-CTGGCCAGATGTTTTCTGGT-3′	5′-TAAAGGGGTCAGCTTTTGG-3′	117
Glil	5′-AACTCCACGAGCACACAGG-3′	5′-GCTCAGGTTTCTCCTCTCTC-3′	79
Smo	5′-GCCTGGTGCTTATTGTGG-3′	5′GGTGGTTGCTCTTGATGG-3′	75
Ptc	5′-AGCGTACCTCCTCCTAGGTAAGCCTC-3′	5′-CGGCTTTATTCAGCATTTCCTC-3′	122
GAPDH	5′-TGTTGCCATCAACGACCCCTT-3′	5′-CTCCACGACATACTCAGCA-3′	202

### Western blotting

Total protein was extracted by homogenizing liver tissue samples of different treatment groups in lysis buffer (50 mM Tris, pH 7.2, 1% Triton X-100, 0.5% sodium deoxycholate, 0.1% SDS, 500 mM NaCl, 10 mM MgCl_2_, with 1 mM PMSF). Then, the proteins were separated by 8-12% SDS-PAGE and transferred onto polyvinylidenedi fluoride membrane. The membranes were blocked with 5% defatted milk and probed with specific primary anti-Vimentin, TGF-β1, BMP-7, Shh, Gli1, Ptc and Smo antibodies (Santa Cruz Biotechnology) or anti-E-cadherin antibody (Abcam), and followed by horseradish peroxidase (HRP)-conjugated goat anti-mouse or rabbit anti-goat IgG secondary antibodies (Santa Cruz Biotechnology). The membranes were then incubated with enhanced chemiluminescent substrate (Santa Cruz Biotechnology) before being exposed to film.

### Statistical analysis

Statistical significance of the data was calculated by One-way ANOVA or Student’s *t*-test. A significance level of *p* < 0.05 was chosen.

## Results

### DWYG treatment inhibited collagen deposition and tissue damage in the livers of rats induced by CCl_4_

**Figure 2:**
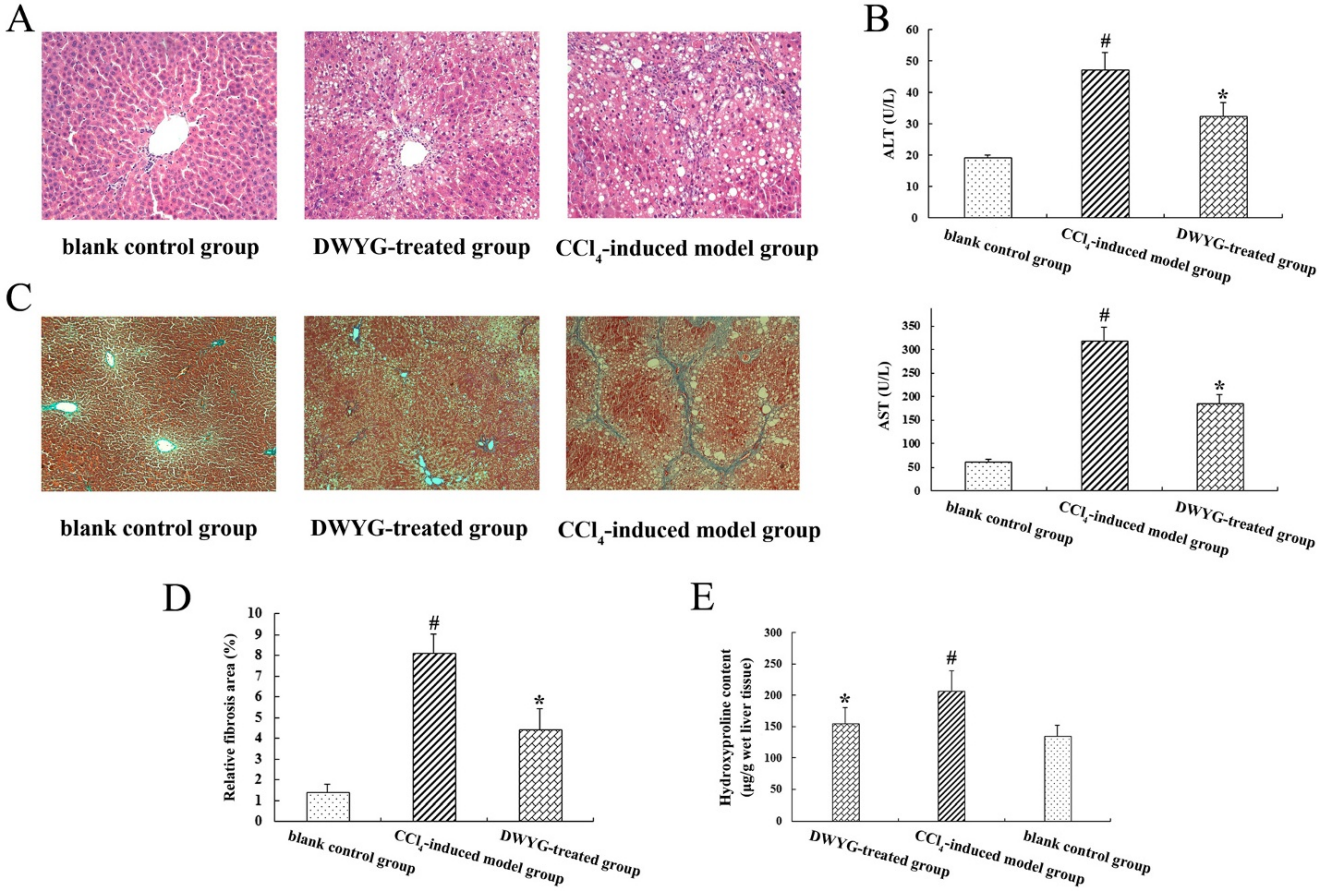
**DWYG treatment inhibited collagen deposition and tissue damage in the livers of rats induced by CCl**_**4**_ **.** Paraffin-embedded liver sections of different treatment groups were stained by H & E **(A)** (original magnification, ×200) and Masson’s trichrome **(C)** (original magnification, ×100) as described in Materials and methods. Representative micrographs are displayed. **(B)** The serum levels of aspartate transaminase (AST) and alanine transaminase (ALT) in different treatment groups were respectively detected by commercial ELISA kits. Data represent the means and standard deviation of 3 independent experiments. #*p* < 0.05 vs. blank control group, *p < 0.05 vs. CCl_4_-induced model group. **(D)** The relative fibrosis areas were evaluated by morphometric analysis of Masson’s trichrome-stained liver tissue sections from different treatment groups. Data represent the means and standard deviation of 3 independent experiments. #*p* < 0.05 vs. blank control group, *p < 0.05 vs. CCl_4_-induced model group. **(E)** Hepatic hydroxyproline content of different treatment groups was assessed as described in Materials and methods. Data represent the means and standard deviation of 3 independent experiments. #*p* < 0.05 vs. blank control group, *p < 0.05 vs. CCl_4_-induced model group.

To evaluate the protective effects of DWYG on CCl_4_-induced liver injury in rats, we examined the histological changes of liver tissues in different treatment groups through H & E staining. As presented in Figure 2A, normal lobular architecture with central veins and radiating hepatic cords was shown in the liver tissue section of blank control groups. Upon chronic CCl_4_ exposure, lobules of liver in model group rats were disorder with a pile of deposition of fibrous tissue, steatosis and necrosis in hepatocytes, whereas DWYG treatment significantly ameliorated the pathological changes observed on CCl_4_-induced injury. Moreover, we detected changes of the serum ALT and AST levels in rats of different treatment groups. As important markers to evaluate liver function, the serum levels of AST and ALT in CCl_4_-induced model group substantially elevated compared with blank control group (Figure 2B, #*p* < 0.05), while DWYG treatment significantly inhibited the increase in serum levels of ALT and AST induced by CCl_4_ (Figure 2B, *p < 0.05). These data suggested that DWYG treatment could ameliorate liver injury and restore liver function.

Chronic CCl_4_ exposure is known to induce progressive fibrogenic liver injury [18]. To investigate the potential impacts of DWYG on the degree of CCl_4_-induced liver fibrosis in rats, we examined the fibrogenic changes of liver tissues in different treatment groups through Masson’s trichrome staining. Figure 2C showed that no fibrous septum was observed in liver tissues of blank control group, whereas fibrous septa became apparent following fibrosis induced by CCl_4_. After DWYG treatment, the degree of liver fibrosis induced by CCl_4_ was dramatically alleviated. Further morphometric analysis based on the Masson’s trichrome staining of liver sections in different treatment groups verified these above observations. As shown in Figure 2D, the relative fibrosis area of CCl_4_-induced model group was obviously increased from 1.41 ± 0.36% to 8.10 ± 0.92% (#*p* < 0.05), whereas the relative fibrosis area of DWYG treatment group was drastically decreased from 8.10 ± 0.92% to 4.41 ± 1.02% (*p < 0.05), indicating that DWYG had an inhibitory effect on the progressive fibrogenic liver injury induced by CCl_4_. Furthermore, we evaluated the hydroxyproline content of liver tissues in different treatment groups. Essentially, all of the hydroxyproline in animal tissues is exclusively found in collagen [19], so the content of hydroxyproline can be considered as an indicator of collagen amount. As indicated in Figure 2E, CCl_4_ induced a considerable elevation in the level of hydroxyproline at 6 weeks (from 135.12 ± 17.74 μg/g wet liver tissue to 207.39 ± 31.66 μg/g wet liver tissue, #*p* < 0.05), while the hydroxyproline content of liver tissues after DWYG treatment was obviously reduced from 207.39 ± 31.66 μg/g wet liver tissue to 155.16 ± 25.22 μg/g wet liver tissue (*p < 0.05). This result was consistent with the inhibitory effect of DWYG on the progressive fibrogenic liver injury based on the histological and morphometric analysis of Masson’s trichrome staining, confirming that DWYG could inhibit the collagen accumulation in liver tissues of CCl_4_-induced fibrotic rats.

### DWYG treatment inhibited EMT and promoted MET in the fibrotic livers of rats induced by CCl_4_

**Figure 3:**
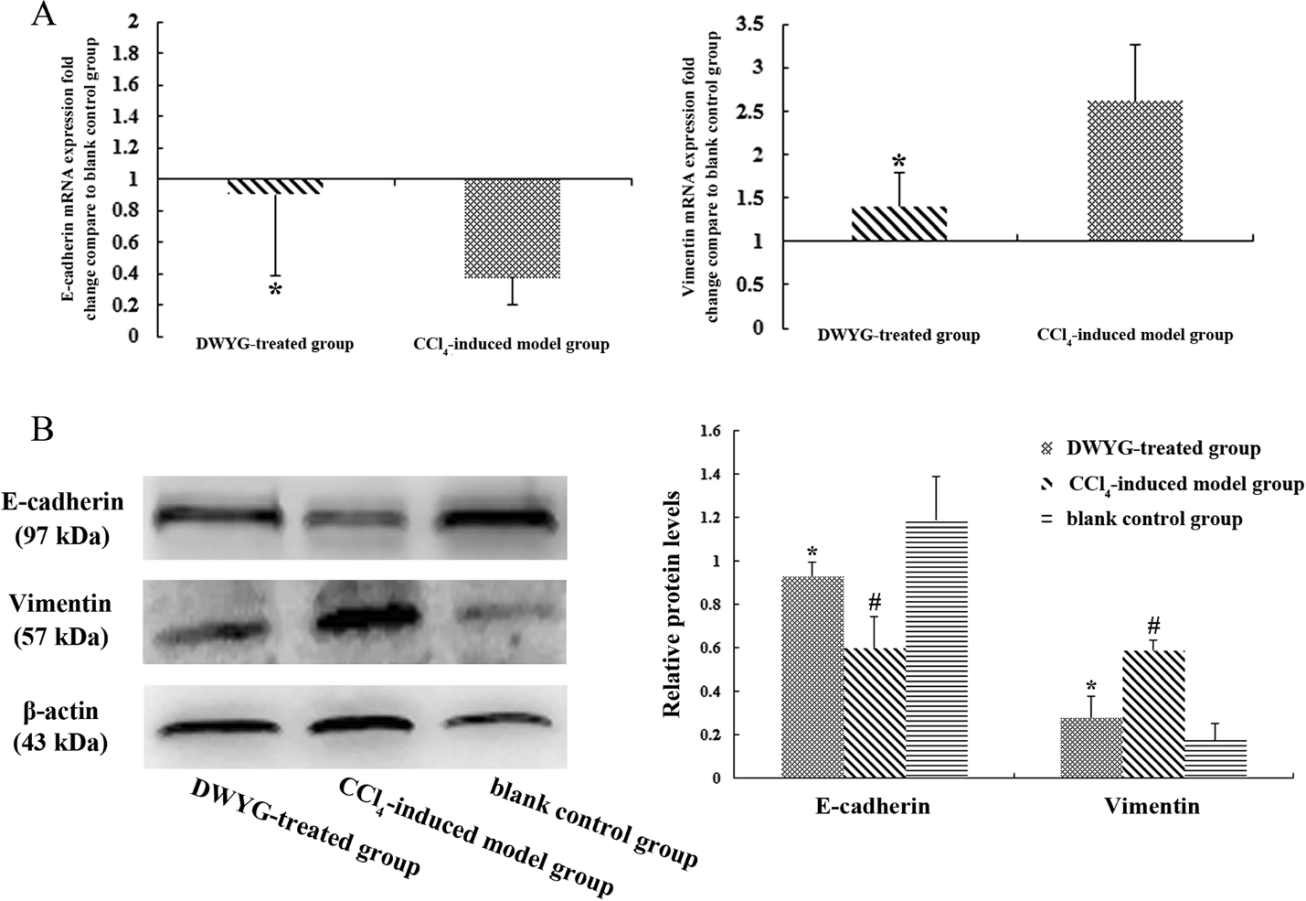
**DWYG treatment inhibited EMT and promoted MET in the fibrotic livers of rats induced by CCl**_**4**_ **. (A)** The mRNA expression levels of E-cadherin and Vimentin in the liver tissues of different treated group rats were detected by quantitative real-time RT-PCR. Delta-delta-CT was calculated as described in Materials and methods, considering GAPDH as internal control and blank control group as reference control. Data represent the means and standard deviation of 3 independent experiments. *p < 0.05 vs. CCl_4_-induced model group. **(B)** The protein expression levels of E-cadherin and Vimentin in the liver tissues of different treated group rats were analyzed by Western blotting. Anti-β-actin blotting was used as control for equal protein loading. Selected blots from one typical experiment are presented. The bar graph represents the densitometric analysis of the bands in 3 independent experiments. #*p* < 0.05 vs. blank control group, *p < 0.05 vs. CCl_4_-induced model group.

To ascertain the potential impacts of DWYG on the EMT and MET in the fibrotic livers of rats induced by CCl_4_, we detected changes in the expression levels of epithelial marker E-cadherin and mesenchymal marker Vimentin by quantitative real-time RT-PCR and Western blot analysis. As indicated in Figure 3A, the mRNA expression levels of E-cadherin and Vimentin in liver tissues of blank control rats were respectively assigned as the baseline levels. After CCl_4_ induction for 6 weeks, the mRNA expression level of E-cadherin in liver tissues of fibrotic model rats was about 60% lower than the baseline level, while the mRNA expression level of Vimentin was about 2.5-fold higher than the baseline level. Consistent with these changes in the mRNA expression level, Western blot analysis also demonstrated that compared with blank control group the content of Vimentin protein in liver tissues of fibrotic model rats increased dramatically, whereas the protein expression of E-cadherin in rat liver tissues virtually disappeared after CCl_4_ induction (Figure 3B, #*p* < 0.05), suggesting that EMT occurred in the evolution of CCl_4_-induced fibrosis. Following treatment with DWYG, the expression pattern of the above epithelial and mesenchymal markers in liver tissues of CCl_4_-induced fibrotic rats was changed. Figure 3A presented that DWYG-treated rats expressed significantly less level of Vimentin mRNA in liver tissues but dramatically greater level of E-cadherin mRNA than those of CCl_4_-induced fibrotic rats (*p < 0.05). Additional Western blot analysis verified that the increase of E-cadherin mRNA in liver tissues of DWYG-treated rats was accompanied by accumulation of the protein, whereas the decrease of Vimentin mRNA was accompanied by diminished protein (Figure 3B, *p < 0.05). These results revealed that DWYG treatment could reverse EMT and promote MET during the progression of fibrosis induced by CCl_4_.

### DWYG treatment recovered the expression ratio of TGF-β1 and BMP-7 in the fibrotic livers of rats induced by CCl_4_

**Figure 4:**
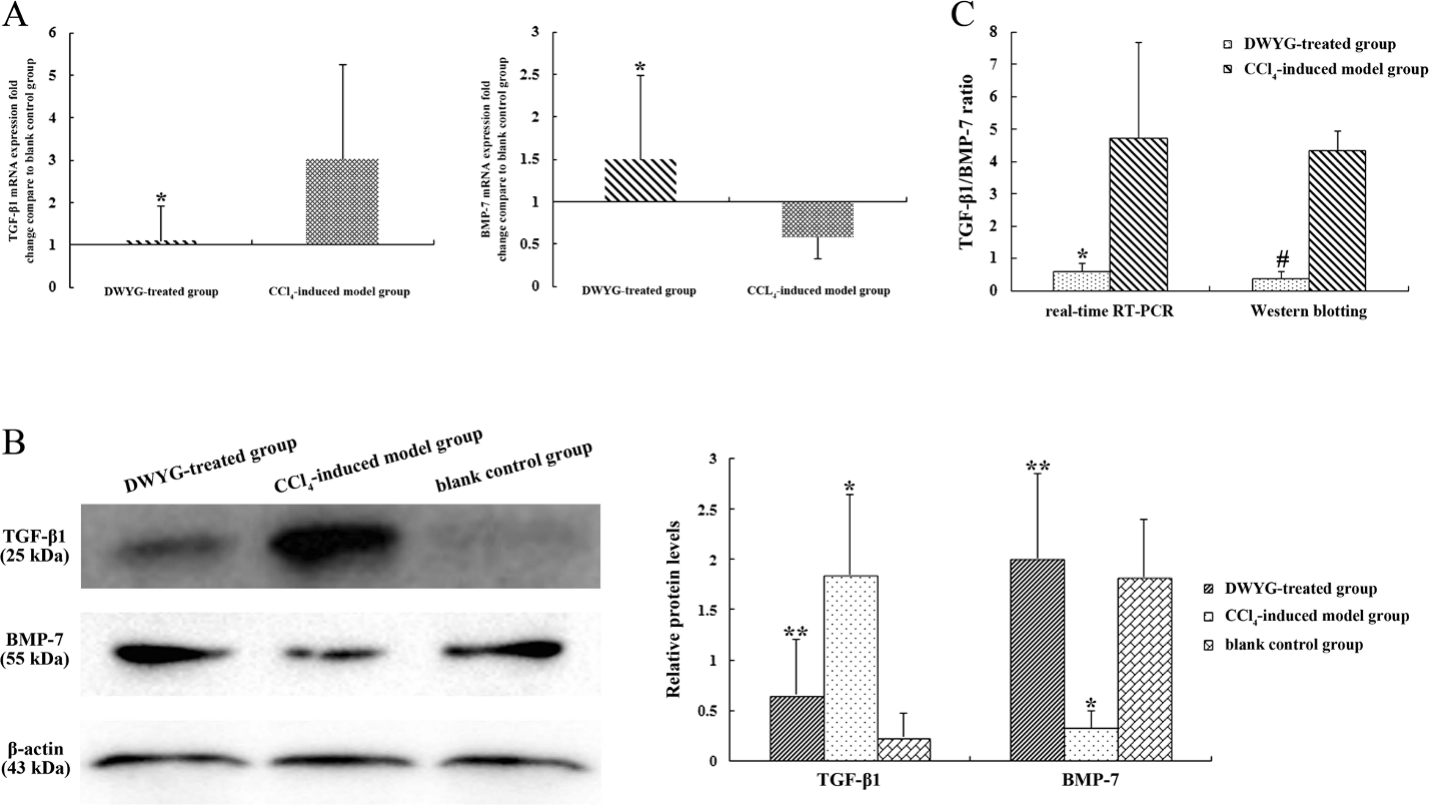
**DWYG treatment recovered the expression ratio of TGF-β1 and BMP-7 in the fibrotic livers of rats induced by CCl**_**4**_ **. (A)** The mRNA expression levels of TGF-β1 and BMP-7 in the liver tissues of different treated group rats were detected by quantitative real-time RT-PCR. Delta-delta-CT was calculated as described in Materials and methods, considering GAPDH as internal control and blank control group as reference control. Data represent the means and standard deviation of 3 independent experiments. *p < 0.05 vs. CCl_4_-induced model group. **(B)** The protein expression levels of TGF-β1 and BMP-7 in the liver tissues of different treated group rats were analyzed by Western blotting. Anti-β-actin blotting was used as control for equal protein loading. Selected blots from one typical experiment are presented. The bar graph represents the densitometric analysis of the bands in 3 independent experiments. *p < 0.05 vs. blank control group, **p < 0.05 vs. CCl_4_-induced model group. **(C)** The mRNA and protein expression ratios of TGF-β1/BMP-7 in the DWYG-treated and CCl4-induced fibrotic liver tissues were statistically analyzed. Data represent the means and standard deviation of 3 independent experiments. *p < 0.05 vs. CCl_4_-induced model group, #*p* < 0.05 vs. CCl_4_-induced model group.

TGF-β1 is believed to play a central role in regulating organ fibrosis. BMP-7, however, a member of the TGF-β1 superfamily, counteracts the fibrogenic action of TGF-β1 [20]. The potential effects of DWYG on TGF-β1 and BMP-7 expression levels in livers of rats induced by CCl_4_ were assessed by quantitative real-time RT-PCR and Western blotting analysis. As shown in Figure 4A, following 6 weeks CCl_4_ induction, the substantial increases of TGF-β1 mRNA and protein expression were observed. Meanwhile, the mRNA and protein expression levels of BMP-7 were dramatically down-regulated in the fibrotic livers. However, DWYG treatment significantly reduced the mRNA expression level of TGF-β1, whereas restored the expression of BMP-7 by stimulating 1.5-fold increase in transcript expression levels (Figure 4A, *p < 0.05). In addition, Western blotting analysis further verified that these changes in TGF-β1 and BMP-7 transcript expression were paralleled by 3-fold decrease in TGF-β1 protein expression and 6.7-fold increase in BMP-7 protein expression after DWYG treatment (Figure 4B, **p < 0.05). A reversed expression ratio of TGF-β1/BMP-7 in fibrotic tissues could be deemed as an anti-fibrotic phenotype [9], thus we further analyze the impact of DWYG on the expression ratio of TGF-β1/BMP-7 in CCl_4_-induced fibrotic liver tissues. Regardless of the mRNA and protein expression levels, the ratio of TGF-β1/BMP-7 in fibrotic livers induced by CCl_4_ was declined by DWYG treatment (Figure 4C, *p < 0.05 and #*p* < 0.05). These opposite effects on the protein and mRNA levels of TGF-β1 and BMP-7 suggested that DWYG treatment could prevent CCl_4_-induced liver fibrosis in rats through recovering the ratio of TGF-β1/BMP-7.

### DWYG treatment inhibited the excessive activation of Hh signaling in the fibrotic livers of rats induced by CCl_4_

**Figure 5:**
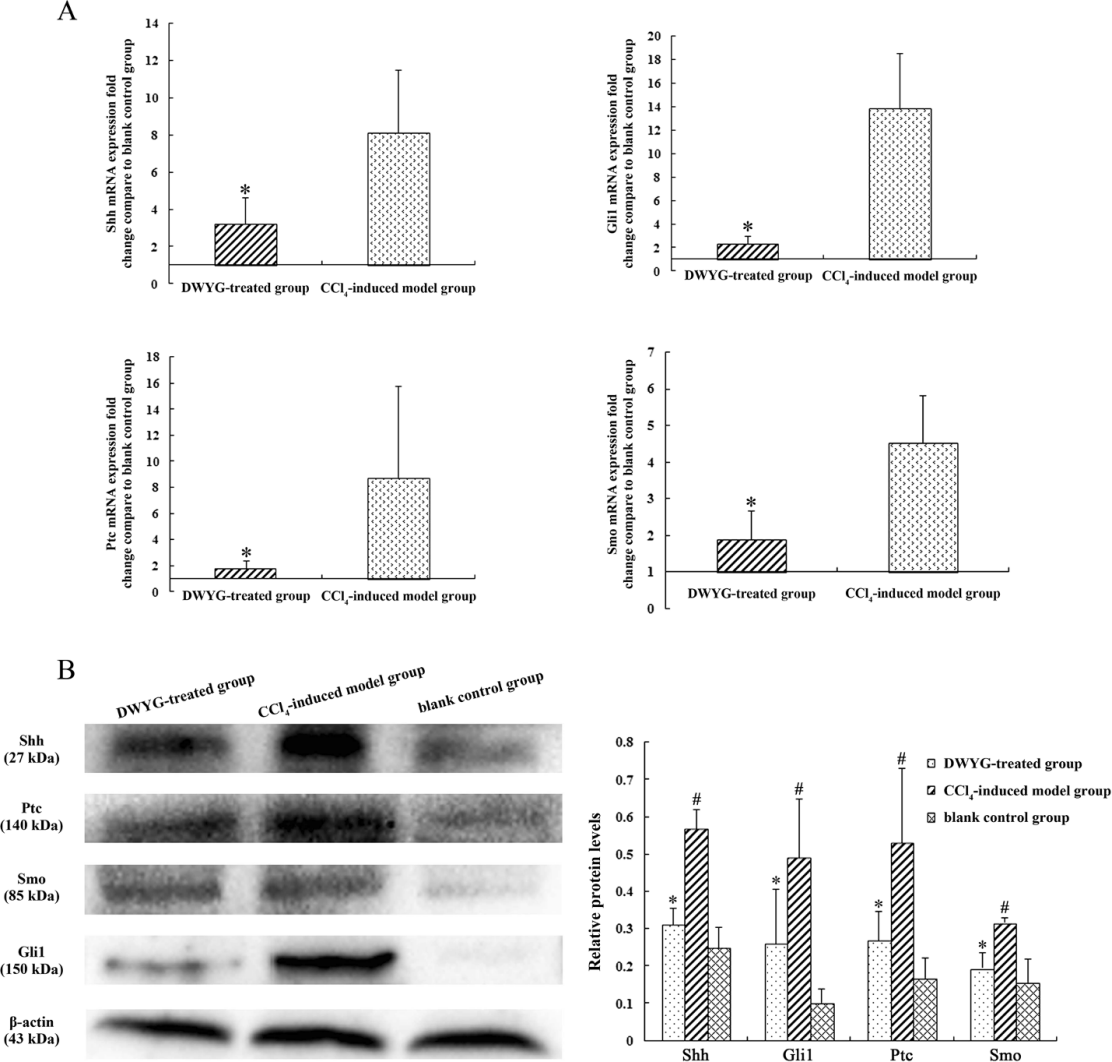
**DWYG treatment inhibited the excessive activation of Hh signaling in the fibrotic livers of rats induced by CCl**_**4**_ **. (A)** The mRNA expression levels of Shh, Gli1, Ptc and Smo in the liver tissues of different treated group rats were detected by quantitative real-time RT-PCR. Delta-delta-CT was calculated as described in Materials and methods, considering GAPDH as internal control and blank control group as reference control. Data represent the means and standard deviation of 3 independent experiments. *p < 0.05 vs. CCl_4_-induced model group. **(B)** The protein expression levels of Shh, Gli1, Ptc and Smo in the liver tissues of different treated group rats were analyzed by Western blotting. Anti-β-actin blotting was used as control for equal protein loading. Selected blots from one typical experiment are presented. The bar graph represents the densitometric analysis of the bands in 3 independent experiments. #*p* < 0.05 vs. blank control group, *p < 0.05 vs. CCl_4_-induced model group.

To assess the mechanism by which DWYG treatment regulate EMT/MET in CCl_4_-induced rat, we focused our investigation on Hh signaling because it has been implicated in the regulation of EMT [21]. Consistent with previously published reports [22], the protein expression of Shh and Gli1 was negligible in liver tissues of blank control rats (Figure 5B). After CCl_4_ induction for six weeks, there was a robust up-regulation of Shh, Smo and Ptc transcript expression concomitant with a substantial up-regulation of Gli1 mRNA compared with blank control group (Figure 5A). Western blot analysis revealed that changes of the above molecules in mRNA expression were accompanied by coincident changes in protein expression (Figure 5B), confirming that Hh signaling pathway was activated in the evolution of liver fibrosis. Moreover, following DWYG treatment, the expression of Shh and Gli1 in mRNA levels drastically reduced compared to that of rats treated with CCl_4_ alone (Figure 5A, *p < 0.05). The down-regulation of these proteins in mRNA expression level was matched by a decrease in hepatic Shh and Gli1 protein expression assessed by Western blot analysis of whole liver tissue (Figure 5B). Additionally, the robust increases of Smo mRNA and protein expression in untreated CCl_4_-induced fibrotic rats were considerably diminished after DWYG treatment. Meanwhile, the expression of its co-receptor Ptc at both mRNA and protein levels were correspondingly diminished (Figure 5B, *p < 0.05). These findings suggested that DWYG treatment could inhibit the excessive activation of Hh signaling in the fibrotic livers of rats induced by CCl_4_.

## Discussion

In recent years, liver fibrosis is gradually regarded as the result of repair and remodel of various liver injuries. The outcome of liver injury is dictated by the effectiveness of repair. Choi SS and Diehl AM put forward the hypothesis that the balance between EMT and MET modulates the outcome of chronic liver injury. When EMT activity outstrips MET, repair is mainly fibrogenic, causing liver fibrosis; conversely, predominance of MET favors more normal liver regeneration [12]. In this study, our data also demonstrated that the fibrogenesis of CCl_4_-induced chronic liver injury accompanied with the occurrence of EMT, one of the key hallmarks of which is loss of E-cadherin; And the treatment with DWYG could result in the reversal of EMT to MET in the fibrotic liver tissue, which was characterized with the up-regulation of E-cadherin expression and down-regulation of Vimentin expression, in accompany with the alleviation of the degree of fibrosis. Herein, the anti-fibrotic mechanism of DWYG is associated with the modulation of the balance between EMT and MET.

Moreover, liver fibrosis is also a complex pathophysiological process involving the multi-link, multi-factor and multi-channel damage [2]. Chemical drugs are generally difficult to achieve significant therapeutic results used alone because of its single therapeutic target [23]. Experimental reports and clinical applications of traditional Chinese medicines against liver fibrosis are in the ascendant due to their effects on multiple targeting and multidirectional regulations [24]. In this study, our data showed that DWYG treatment could modulate the balance between EMT and MET through at least two different mechanisms: reducing the expression ratio of TGF-β1/BMP-7 and inhibiting the excessive activation of Hh signaling pathway.

TGF-β1, as one of the key mediators of fibrogenesis, is considered as the master regulator of EMT. TGF-β1 can induce EMT in various types of cultured nonmaliganant and malignant epithelial cells, including hepatocyte and cholangiocyte, through different signaling mechanisms [20]. Our data also demonstrated that the expression level of TGF-β1 in the CCl_4_-induced fibrotic liver tissue was dramatically elevated, and then obvious EMT was provoked in the fibrotic liver tissue. Moreover, BMP-7, a member of the TGF-β surperfamily that antagonizes TGF-β1 signaling, negatively regulates TGF-β1-induced EMT in different types of organ injury, including liver fibrosis. Both pharmacological administration and genetic expression of BMP-7 have been shown not only to prevent organ fibrotic injury by inhibiting TGF-β-provoked EMT but also to promote organ recovery by stimulating the re-population of injured tissues with healthy cells, partly through inducing MET [25, 26]. As evidenced by our data, the up-regulated expression level of BMP-7 induced the occurrence of MET in the liver tissues of DWYG-treated rats, which was characterized with the up-regulated expression level of E-cadherin. These results are consistent with previous study [9], indicating that TGF-β1 signaling tends to promote EMT, whereas BMP-7 signaling seems not only to counteract EMT, but also to promote MET, even in adult tissue. Therefore, the expression ratio of TGF-β1/BMP-7 could be considered as the regulator factor to modulate the balance between EMT and MET. Our data also demonstrated that DWYG could obviously decrease the expression ratio of TGF-β1/BMP-7 in CCl_4_-induced fibrotic liver tissue, which facilitated the reversal of EMT to MET in liver tissues and attenuated the degree of liver fibrosis.

Like TGF-β1/BMP-7, Hh signaling also modulates EMT/MET in adult liver repair and regeneration. Hh signaling is a key morphogenetic pathway that controls fetal liver development and also plays an essential role in the modulation of wound healing responses in many types of adult liver injury [22]. Hh signaling pathway is quiescent in normal liver, but becomes reactivated as a repair mechanism in chronic liver injury. However, excessive or persistent Hh pathway activity actually aborts successful regeneration of damaged liver tissue and contributes to the pathogenesis of liver fibrosis by promoting EMT (while inhibiting MET) [22]. Our study showed that chronic exposure to CCl_4_ activated the Hh signaling pathway, which was manifested as the elevated mRNA and protein levels of Hh ligand Shh and transcription factor Gli1. Meanwhile, during the course of CCl_4_-induced liver fibrogenesis, the increases of hepatic collagen content and EMT markers expression in liver tissues of fibrotic model rats were paralleled by the elevation of Hh pathway activity, which also proved that the dysregulation of Hh signaling pathway plays a pivotal role in modulating liver injury and initiation of EMT that accelerates the induction of fibrosis [7, 27]. After DWYG treatment, the expressions of Hh ligand Shh, membrane-spanning receptor Smo and its co-receptor Ptc, and transcription factor Gli1 in CCl_4_-induced fibrotic liver tissue were dramatically repressed, suggesting that the activity of Hh signaling pathway was inhibited. The inhibition of Hh signaling pathway restored the expression of epithelial marker E-cadherin, repressed the expression of mesenchymal marker Vimentin and provoked the reversal of EMT to MET in CCl_4_-induced fibrotic liver tissue, which contributed to alleviating the degree of liver fibrosis.

## Conclusions

We described the antifibrotic effect of DWYG and its possible mechanisms on CCl_4_-induced fibrotic model rats. Importantly, our study not only underscored that the modulation of the balance between EMT and MET (inhibiting EMT while promoting MET) is a novel strategy for prevention of fibrotic diseases, but also set a foundation for the rational utilization of DWYG in combating liver fibrosis. Moreover, multiple mechanisms, including reducing the expression ratio of TGF-β1/BMP-7 and inhibiting the excessive activation of Hh signaling pathway, were involved in modulating the balance between EMT and MET by DWYG treatment, which further demonstrate the advantage of traditional Chinese medicine compound with multi-component and multi-target in the therapy for liver fibrosis.
